# Prevalence and Factors Associated with Hepatitis B Virus Infection Among Senior Citizens in a Southern Brazilian City

**DOI:** 10.5812/hepatmon.7874

**Published:** 2013-04-30

**Authors:** Danúbia Felippe Grassi de Paula Machado, Tatiana Martins, Daisson José Trevisol, Roger Augusto Vieira e Silva, Janaína Luz Narciso-Schiavon, Fabiana Schuelter Trevisol, Leonardo de Lucca Schiavon

**Affiliations:** 1 Post-Graduate Program in Health Sciences, University of Southern Santa Catarina, Tubarão, Santa Catarina, Brazil; 2 Tubarão City Hall, Santa Catarina, Brazil; 3 Division of Gastroenterology, Federal University of Santa Catarina, Santa Catarina, Brazil

**Keywords:** Hepatitis B, Epidemiology, Infectious Disease Transmission, Prevalence, Risk Factors

## Abstract

**Background:**

Given the long term exposure to risk factors, it is likely that older adults exhibit the highest proportions of HBV serological markers. Nevertheless, there are few methodologically adequate studies in Brazil evaluating the prevalence and risk factors for HBV infection in individuals aged 60 years or more.

**Objectives:**

To estimate the prevalence and factors associated with HBV infection in elderly residents in the city of Tubarão/SC.

**Patients and Methods:**

This cross-sectional study included 820 individuals (≥ 60 years) selected by simple random sampling. The variables were compared by chi-square test or Fisher's exact test and those with P < 0.200 were included in the regression model.

**Results:**

The mean age of patients was 68.6 ± 7.0 years, 39% were men and 92% Caucasian. Five subjects (0.6%) presented with positive HBsAg and 124 (15.1%) were anti-HBc reactive. Bivariate analysis showed that the presence of anti-HBc was associated with age ≥ 67 years, ≤ 4 years of schooling, acupuncture therapy and lower proportion of subjects exposed to invasive procedures. In multivariate analysis, the following variables remained independently associated with HBV infection: male gender, marital status, ≤ 4 years of schooling and acupuncture.

**Conclusions:**

The prevalence of anti-HBc among the elderly in the city of Tubarão was higher than in previous studies evaluating blood donors in the same region. Despite the association of previous HBV infection and factors indirectly related to sexual risk behaviors, the results suggest the involvement of invasive therapeutic procedures in the HBV transmission chain.

## 1. Background

Hepatitis B virus (HBV) infection is a major public health problem. About two billion people worldwide have been infected and more than 350 million are chronic HBV carriers (1). HBV infection is transmitted by parenteral route, by percutaneous or permucosal exposure to blood or other body fluids (2). In low endemic regions, most cases of hepatitis B are related to sexual exposure, while in high endemic areas perinatal or early childhood transmission is the most common form of HBV infection (2). The global prevalence of chronic HBV infection is about 5%, but it varies significantly according to the geographic region. Latin America is usually considered an area of low prevalence; nevertheless, there is a great regional variation, with high endemicity observed in the Amazon region and in specific subpopulations (3). Evidence suggests a higher prevalence of HBV infection markers among the elderly (4, 5), probably reflecting a longer exposure to risk factors. However, another possible explanation would be a real change in the epidemiologic profile of HBV infection in a similar way to that observed in the human immunodeficiency virus (HIV) infection, with a displacement of the prevalence curve for higher age groups (4). Besides the higher prevalence, the elderly present peculiarities that negatively influence various aspects of HBV infection, such as higher rates of progression to chronic forms after acute HBV infection and, when chronic carriers, they are most likely to present more advanced liver disease (5-8). Data regarding the epidemiological characteristics of HBV infection in Brazil and South America are scarce and, as most studies included blood donors, the elderly are often underrepresented.

## 2. Objectives

The aim of this study was to estimate the prevalence and investigate possible factors associated with HBV infection (current or past) in the elderly in the municipality of Tubarão, southern Brazil.

## 3. Patients and Methods

### 3.1. Study Population

This observational cross-sectional population-based study was conducted between June 2010 and March 2011 in the municipality of Tubarão, located in the state of Santa Catarina, southern Brazil. Individuals aged ≥ 60 years (completed in 2010) enrolled in the Program of Family Health Strategy (FHS) were included. Those who refused or declined to participate in the study and those who did not attend the blood collection unit were excluded (apart from those with mobility problems for whom samples were taken at their home). The FHS program is a strategic initiative of the Brazilian Public Health System which involves multidisciplinary teams, and aims to promote health, prevention, recovery and rehabilitation of diseases. The FHS Program of Tubarão has a good coverage rate (> 75%), totaling 9,009 elderly subjects in June 2010. Assuming an estimated prevalence rate of 1.8% of total anti-HBc in the population (9), with a 95% confidence interval and accuracy of 0.9%, the estimated sample size was 781 individuals (10). In anticipation of possible losses, 234 patients (30%) were additionally included, totaling 1015 patients who were selected by simple random sampling. A pre-survey was conducted on all elderly subjects enrolled in the health units, and the data was entered into a spreadsheet to perform the random sampling that was generated by the Random Generator of the Microsoft® Excel® software (Add-in Express Ltd). The study protocol conformed to the ethical guidelines of the 1975 Helsinki Declaration and was approved by our institutional review board.

### 3.2. Data Collection

The individuals were interviewed at home after being informed about the objectives of the study, the assurance of data confidentiality and the approval of the informed consent form. The structured questionnaire consisted of closed questions about sociodemographic characteristics, medical and surgical history and risk behaviors, including drug use and sexual practices. Alcoholism was defined by the finding of two or more affirmative responses to the CAGE questionnaire (11). Those who had reported to smoke 100 or more cigarettes in their lifetime were considered smokers (current or previous) (12). In the present study, invasive procedures were defined as all operative procedures in which skin or mucous membranes were incised, or an instrument was introduced through a natural body orifice. This definition includes all surgical procedures; minimally invasive dermatological interventions; techniques such as percutaneous transluminal angioplasty and cardiac catheterization; minimally invasive procedures involving biopsies or placement of probes or catheters requiring entry into a body cavity through a needle or trocar; and endoscopic procedures. After the interview, participants were scheduled for blood collection in one of the FHS units. Home collections were performed in cases of individuals with limited mobility. The samples were centrifuged in loco by the collection team and transported to the Clinical Laboratory of Unisul for testing. In the case of household blood collection, the samples were transported immediately to the laboratory and then centrifuged and processed.

### 3.3. Laboratory Analysis

HBsAg and total anti-HBc were tested by enhanced chemiluminescence (Vitros. Eci, Johnson & Johnson, USA). According to the results of these tests, the individuals could be found in one of the following categories: 1 - Negative for HBV markers (all tests negative), 2 - Current HBV infection (HBsAg positive and anti-HBc reactive or not) and 3 - Previous infection with HBV (HBsAg negative and anti-HBc reactive). Indeterminate anti-HBc results were treated as negative for the evaluation of factors associated with HBV infection.

### 3.4. Statistical Analysis

Numerical variables were expressed as mean and standard deviation, whereas categorical variables were described in absolute numbers and proportions. Pearson’s chi-square and Fisher’s exact tests were used for comparison of proportions. P value of less than 0.050 was considered as statistically significant. In order to identify parameters independently associated with HBV infection, the variables with P < 0.200 in the bivariate analysis were evaluated by logistic regression using the Enter method. The goodness-of-fit of the final logistic regression model was verified by the Hosmer-Lemeshow test (P value > 0.050 indicated that the model had appropriate adjustment). A P-value < 0.050 was considered statistically significant. All tests were performed by the SPSS software, version 17.0 (SPSS, Chicago, IL, USA).

## 4. Results

### 4.1. Characteristics of the Sample

Of the 1015 subjects selected for inclusion, 122 did not attend the interview for the following reasons: eight died, five moved to other cities, 11 were not located and 98 refused to participate. Of the 893 who were interviewed, 73 did not attend the blood collection unit and were excluded from the final analysis. The characteristics of the included subjects and the comparison with excluded individuals are exhibited in Tables 1 and 2. The mean age of the included elderly was 68.7 ± 7.0 years; 38.5% were men and 92.4% were Caucasian. When compared to excluded subjects, those included in the analysis showed a higher proportion of stable relationships (married or cohabiting) and were less prone to share personal items with non-household members. No differences were observed in the comparison of other variables.

**Table 1.** Comparison Between Included (n = 820) and Excluded Subjects (n = 73) RegardingSociodemographic Characteristics

Characteristics	Included, No. (%)	Excluded, No. (%)	P value ^a^
**Sex**			0.244
**Male**	316 (38.5)	23 (31.5)	
**Female**	504 (61.5)	50 (68.5)	
**Age, y**			0.142
**60-67**	433 (52.8)	32 (43.8)	
**> 67**	387 (47.2)	41 (56.2)	
**Self-reported race**			0.343
**Caucasian**	756 (92.4)	65 (89.0)	
**Non-Caucasian**	62 (7.6)	8 (11.0)	
**Schooling, y**			0.255
**0-3**	398 (48.5)	30 (41.7)	
**≥ 4**	422 (51.5)	42 (58.3)	
**Stable relationship**			0.007
**Yes**	541 (66.0)	36 (49.3)	
**No**	279 (34.0)	37 (50.7)	
**Members in the household**			0.881
**0-2**	467 (57.0)	41 (56.2)	
**≥ 3**	353	32 (43.8)	

^a^chi-square test

**Table 2.** Comparison Between Included (n = 820) and Excluded Subjects (n = 73) Regarding Variables Related to Exposure and Risk Factors

Characteristics	Included, No. (%)	Excluded, No. (%)	P value ^a^
**Alcoholism**			0.911
**Yes**	83 (10.1)	7 (9.6)	
**No**	737 (89.9)	66 (90.4)	
**Current/previous smoking**			0.271
**Yes**	331 (40.6)	34 (47.2)	
**No**	485 (59.4)	38 (52.8)	
**History of drug use**			1.000 ^b^
**Yes**	3 (0.4)	0 (0)	
**No**	815 (99.6)	71 (100)	
**Regular use of condoms**			0.210 ^b^
**Yes**	18 (2.2)	3 (4.5)	
**No**	795 (97.8)	64 (95.5)	
**Sexual partners ** ^c^			0.416 ^b^
**0-1**	793 (97.5)	66 (95.7)	
**≥ 2**	20 (2.5)	3 (4.3)	
**Blood transfusion**			0.173
**Yes**	129 (15.8)	7 (9.7)	
**No**	690 (84.2)	65 (90.3)	
**Blood transfusion < 1993**			0.262
**Yes**	64 (7.9)	3 (4.2)	
**No**	745 (92.1)	68 (95.8)	
**Invasive procedures**			0.671
**Yes**	497 (60.8)	42 (58.3)	
**No**	321 (39.2)	30(41.7)	
**Acupuncture**			0.413
**Yes**	68 (8.3)	8 (11.1)	
**No**	751 (91.7)	64 (88.9)	
**Tattoo**			0.225 ^b^
**Yes**	2 (0.2)	1 (1.4)	
**No**	813 (99.8)	71 (98.6)	
**Sharing personal items**			0.550
**Yes**	249 (30.5)	24 (33.8)	
**No**	567 (69.5)	47 (66.2)	
**Sharing items with household members**			0.226
**Yes**	138 (16.9)	8 (11.3)	
**No**	677 (83.1)	63 (88.8)	
**Sharing items with non-household members**			0.023
**Yes**	123 (15.1)	18 (25.4)	
**No**	692 (84.9)	53 (74.6)	
**Previous HIV testing**			0.560
**Yes**	74 (9.1)	5 (7.0)	
**No**	739 (90.9)	66 (93.0)	
**Previous vaccination for HBV**			0.706
**Yes**	69 (8.4)	7 (9.7)	
**No**	750 (91.6)	65 (90.3)	

^a^Chi-square test

^b^Fisher’s Exact Test

^c^In the past twelve months

### 4.2. Prevalence and Factors Associated With HBV Infection

**Figure 1.**
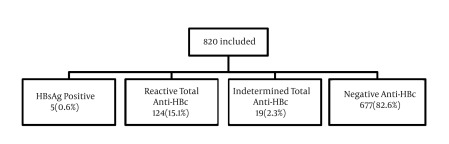
Flowchart Detailing the Studied Sample According to the HBV Serological Pattern. All Subjects With Positive HBsAg had Reactive Total Anti-HBc

As shown in Figure 1, the HBsAg was positive in five subjects (0.6%), total anti-HBc was reactive in 124 (15.1%) and indeterminate in 19 (2.3%) individuals. All HBsAg positive patients were also reactive for anti-HBc.

In the study sample, 677 subjects (82.6%) had no markers of HBV infection. Therefore, in this study, 124 (15.1%) of the elderly were considered individuals with current or previous HBV infection. In the bivariate analysis of sociodemographic variables, those with evidence of HBV infection had a higher proportion of individuals over 67 years of age (56.5% vs. 45.5%, P = 0.025) and a lower proportion of elderly people with four or more years of schooling (40.3% vs. 53.4%, P = 0.007) (Table 3). Concerning the variables related to exposure and risk factors (Table 4), anti-HBc reactive subjects showed a higher proportion of patients previously submitted to acupuncture (13.7% vs. 7.3%, P = 0.018) and a lower proportion of previous invasive procedures (51.6% vs. 62.4%, P = 0.024). No differences were observed in the comparison of other variables.

**Table 3.** Sociodemographic Characteristics of the 820 Elderly Patients Included in the Analysis and Their Association With HBV Infection (Current or Past)

Characteristics	HBV (+), No. (%)	HBV (-), No. (%)	P value ^a^
**Sex**			0.100
**Male**	56 (45.2)	260 (37.4)	
**Female**	68 (54.8)	436 (62.6)	
**Age, y**			0.025
**60-67**	54 (43.5)	379 (54.5)	
**> 67**	70 (56.5)	317 (45.5)	
**Self-reported race**			0.219
**Caucasian**	117 (95.1)	639 (91.9)	
**Non-caucasian**	6 (4.9)	56 (8.1)	
**Schooling, y**			0.007
**0-3**	74 (59.7)	324 (46.6)	
**≥ 4**	50 (40.3)	372 (53.4)	
**Stable relationship**			0.161
**Yes**	75 (60.5)	466 (67.0)	
**No**	49 (39.5)	230 (33.0)	
**Members in the household**			0.639
**0-2**	73 (58.9)	394 (56.6)	
**≥ 3**	51 (41.1)	302 (43.4)	

^a^chi-square test

**Table 4.** Variables Related to Exposure and Risk Factors of the 820 Elderly Patients Included in the Analysis and Their AssociationWith HBV Infection (Current or Past)

Characteristics	HBV (+) No. (%)	HBV (-) No. (%)	P value ^a^
**Alcoholism**			0.270
**Yes**	16 (12.9)	67(9.7)	
**No**	108 (87.1)	627 (90.3)	
**Current/previous smoking**			0.462
**Yes**	54 (43.5)	227 (40.0)	
**No**	70 (56.5)	415 (60.0)	
**History of drug use**			0.390 ^b^
**Yes**	1 (0.8)	2 (0.3)	
**No**	123 (99.2)	692 (99.7)	
**Regular use of condoms**			0.466 ^b^
**Yes**	2 (1.6)	16 (2.3)	
**No**	122 (98.4)	673 (97.7)	
**Sexual partners ** ^c^			0.364 ^b^
**0-1**	120 (96.8)	673 (97.7)	
**≥2**	4 (3.2)	16 (2.3)	
**Blood transfusion**			0.682
**Yes**	18 (14.5)	111(16.0)	
**No**	106 (85.5)	584 (84.0)	
**Blood transfusion < 1993**			0.249
**Yes**	13 (10.5)	51 (7.4)	
**No**	111 (89.5)	634 (92.6)	
**Invasive procedures**			0.024
**Yes**	64 (51.6)	433 (62.4)	
**No**	60 (48.4)	261(37.6)	
**Acupuncture**			0.018
**Yes**	17 (13.7)	51 (7.3)	
**No**	107 (86.3)	644 (92.7)	
**Tattoo**			0.719 ^b^
**Yes**	-	2 (0.3)	
**No**	124 (100.0)	689 (99.7)	
**Sharing personal items**			0.921
**Yes**	38 (30.9)	211 (30.4)	
**No**	85 (69.1)	482 (69.6)	
**Sharing items with household members**			0.162
**Yes**	26 (21.3)	112 (16.2)	
**No**	96 (78.7)	581 (73.8)	
**Sharing items with non-household members**			0.138
**Yes**	13 (10.7)	110 (15.9)	
**No**	109 (89.3)	583 (84.1)	
**Previous HIV testing**			0.923
**Yes**	11 (8.9)	63 (9.1)	
**No**	113 (91.1)	626 (90.9)	
**Previous vaccination for HBV**			0.370
**Yes**	13 (10.5)	56 (8.1)	
**No**	111 (89.5)	639 (91.9)	

^a^chi-square test

^b^fisher’s exact test

^c^in the past twelve months

Multiple logistic regression analysis was performed using HBV infection (current or past) as the dependent variable. The following variables with P < 0.200 were included in the regression model: gender, age dichotomized at 67 years, education dichotomized in four years, marital status, history of invasive procedures, history of acupuncture, sharing personal items with household members/non-household members. In the multivariate analysis (Table 5), the variables that were independently associated with HBV infection were male gender (OR 1.674, 95% CI 1.064 to 2.634, P = 0.026), schooling < 4 years (OR 1.532, 95% CI 1.024 to 2.292, P = 0.038), no stable relationships (OR 1.577, 95% CI 1.002 to 2.483, P = 0.049) and previous acupuncture therapy (OR 2.520, 95% CI 1.360 to 4.668, P = 0.003).

**Table 5.** Logistic Regression Analysis of the Factors Associated With the Presence of HBV Infection (VariablesWith P < 0.200 in the Bivariate Analysis)

Factors	Odds Ratio	CI ^a^	P value
**Male gender**	1.674	1.064 – 2.634	0.026
**Age > 67 years**	1.315	0.873 – 1.980	0.190
**Schooling < 4 years**	1.532	1.024 – 2.292	0.038
**No stable relationship**	1.577	1.002 – 2.483	0.049
**History of Invasive Procedures**	0.722	0.482 – 1.082	0.115
**Acupuncture**	2.520	1.360 – 4.668	0.003
**Sharing items with household members**	1.533	0.934 – 2.518	0.091
**Sharing items with non-household members**	0.750	0.395 – 1.423	0.379

^a^Abbreviations: CI, Confidence Interval

## 5. Discussion

The present work represents the first population-based study in a Brazilian city with the aim of determining the prevalence of HBV infection in elderly. Even if we consider Brazilian studies on the prevalence of Hepatitis B in general, most of them had significant limitations, and were generally carried out in small groups and were not representative of the general population (e.g. blood donors). The prevalence of current HBV infection in this study was 0.6% and the prevalence of markers of previous contact was 15.1%. With regards to HBsAg, these results are similar to studies with blood donors, which showed a prevalence of 0.64% and 0.78% for the state of Santa Catarina and the city of Tubarão, respectively (9). However, the prevalence of anti-HBc in this study was significantly higher than that observed in blood donors of the same region (5.35% and 3.92%, for the state of Santa Catarina and the municipality of Tubarão, respectively) (9). This divergence suggests a higher prevalence of the marker of contact between the elderly, but it may also reflect other specific characteristics of blood donors. In 2011 data from the largest study ever, conducted in Brazil on the prevalence of viral hepatitis was released (13). This study included 19,634 individuals aged 10 to 69 years, residents of the Brazilian state capitals. The prevalence of current HBV infection, among adults was 0.6% and 11.6% exhibited serological profiles compatible with previous HBV infection. The results remained similar when only residents of the South region were evaluated, with 0.55% prevalence for HBsAg and 11.3% for anti-HBc. Although the proportion of HBsAg positive was similar in the present study, a higher prevalence of anti-HBc was observed among the elderly in the city of Tubarão. In fact, when only the age group between 60 and 69 years in a Southern population-based study of the Ministry of Health is taken into account, the prevalence of positive anti-HBc was 23%, which is significantly higher than that found for younger individuals. It is likely that in the elderly, the longer exposure to risk factors justifies a higher prevalence of markers of HBV infection, however, the longer duration of the infection may also justify a higher probability of seroconversion to anti-HBs throughout life, explaining the dissociation between the numerical proportions of individuals with markers of past and current infection among the elderly (14, 15). In the logistic regression analysis, male gender, no stable relationship, lower education (< 4 years) and previous acupuncture therapy were independently associated with HBV infection. Despite the association observed in the univariate analysis, age over 67 years and a history of non-invasive procedures did not remain in the final model of the logistic regression. Regarding the higher prevalence of HBV infection in males, several studies from different regions of the world and with different methodologies are consistent with these finding (16-21). The association between male gender and hepatitis B is probably the result of greater exposure to risky sexual behavior among men, a hypothesis reinforced by the higher prevalence of other sexually transmitted diseases among these individuals (22). The population-based study of the Brazilian Ministry of Health assessed the factors associated with positive anti-HBc in different regions (13). No differences were observed with regards to gender in the North and South, however, in the other regions, multivariate analysis showed an association between male and reactive anti-HBc. It is likely that peculiarities of HBV epidemiology in the South and North justify the differences reported in the study of the Ministry of Health. The high endemicity observed in the North (especially in the Amazonas state) and also in the western states of the South Region (especially Santa Catarina) may be related to an increased number of cases of vertical transmission, thus indirectly reducing the impact of sexual transmission as a route of HBV transmission in the capital cities of these regions, which often receive people coming from endemic areas (9, 23). The association between the male gender and HBV infection observed in the present study probably reflects regional characteristics, the greatest distance from most endemic areas and the absence of well-defined population flow from the west of the State to the city of Tubarão justify higher rates of sexual transmission. Emphasizing the importance of variables associated with sexual exposure, in the present study the presence of anti-HBc was independently associated with the absence of a stable relationship. This finding is in agreement with that observed in previous studies (24, 25) and, as discussed above, is probably indirectly related to sexual risk behaviors. Interestingly, other variables usually more directly related to sexually transmitted diseases, such as a greater number of sexual partners in the last twelve months and regular use of condoms, were not associated with HBV infection. This is justified by the fact that probably the majority of HBV infections in this group of individuals occurred in the distant past. Thus, these variables are related to characteristics of more recent sexual behavior, which do not necessarily reflect the situation at the time of HBV contamination. Furthermore, from a statistical point of view, the low frequency observed for these variables may have significantly limited the ability to identify more subtle differences.

Lower education was independently associated to the presence of HBV infection. The association between hepatitis B and lower education has been previously demonstrated in international studies (24) and in Brazilian blood donors (26). Similarly, the population-based study of the Ministry of Health found similar results in all regions of Brazil, with the exception of the Southeast (13). Specifically in the South, higher education (high school or college) was associated with lower prevalence of anti-HBc. Schooling is one indicator of socioeconomic status and these results probably reflect that a greater exposure to HBV occurs in situations of greater poverty and less access to information regarding preventive measures. In the present study, the multivariate analysis showed an association between previous acupuncture therapy and HBV infection. Similarly to other invasive procedures, failure or non-adoption of precautionary measuremay be associated with risk of contamination by parenterally transmitted infectious agents such as HBV. In fact, previous reports, particularly Asian studies from countries with high HBV endemicity, demonstrated an association between acupuncture and HBV infection.

(27-31). Although other authors have not reported this association (32, 33), the findings of this study and others reinforce the need for control over the institutions and professionals involved in invasive procedures, ensuring the adoption of precautionary measures in order to prevent the nosocomial contamination by parenterally transmitted agents. Moreover, it is important to implement strategies to ensure adequate vaccine coverage against hepatitis B among healthcare workers, especially those who engage frequently in situations with increased risk of contamination.

Exposure to other invasive procedures was not associated with the presence of HBV infection in the elderly. It is possible that these findings are related to the high proportion of individuals previously subjected to procedures that were considered invasive. Thus, it is likely that a better detailed variable, including the type of procedure (in- or out-hospital), the magnitude or extend (small, medium and large) and the health professionals involved (health professionals or not) would provide important information about this risk factor. Some limitations of this study should be discussed. First, the possibility of occult HBV infection, especially in those with the anti-HBc reactive/HBsAg negative profile, was not investigated in the present study. Although this could affect the prevalence of “true” current HBV infection, the implications of this information in epidemiological studies is still a matter of discussion. In addition, the exploration of occult HBV infection was outside the scope of this investigation and the methodology used in the present study are in agreement with the majority of epidemiology studies on HBV infection. Secondly, the differences between included and excluded individuals regarding marital status and the proportion of subjects who share personal items with non-household members may suggest selection bias. However, this is unlikely to have occurred as the proportion of excluded subjects was relatively small and they were similar to included ones regarding all other major characteristics. Finally, the study design adopted does not allow establishment of direct causal relationships and the temporal sequence between the variables investigated and HBV infection. In fact, this is a common limitation of cross-sectional studies and can be resolved with the cohort studies (34). Nevertheless, cross-sectional population studies represent useful tools for public health planning, understanding of disease etiology and hypothesis generation (35).

In conclusion, the prevalence of current HBV infection (HBsAg) among the elderly population of the municipality of Tubarão was 0.6%, which is similar to that described in blood donors from the same city. However, the proportion of subjects with reactive anti-HBc was 15.1%, which is substantially higher than that observed in blood donors. It is more likely that these findings reflect a cumulative effect of exposure to risk factors throughout life than a change in the epidemiological profile with recent exposure to HBV. This hypothesis is justified by the wide prevalence of the anti-HBc reactive/HBsAg negative profile (previous infection) in relation to the anti-HBc reactive/HBsAg positive profile (current infection). The presence of HBV infection was independently associated with male gender, no stable relationship, lower educational level and history of acupuncture. These findings reflect the importance of factors indirectly related to sexual risks and low socioeconomic status, but also suggest the possible involvement of invasive therapeutic procedures in the HBV transmission chain.

## References

[1] Lavanchy D (2004). Hepatitis B virus epidemiology, disease burden, treatment, and current and emerging prevention and control measures.. J Viral Hepat..

[2] Liaw YF, Chu CM (2009). Hepatitis B virus infection.. Lancet..

[3] Parana R, Almeida D (2005). HBV epidemiology in Latin America.. J Clin Virol..

[4] Nguyen N, Holodniy M (2008). HIV infection in the elderly.. Clin Interv Aging..

[5] Chiaramonte M, Floreani A, Naccarato R (1987). Hepatitis B virus (HBV) in the elderly: an underestimated problem?. Biomed Pharmacother..

[6] Hadziyannis SJ, Papatheodoridis GV, Dimou E, Laras A, Papaioannou C (2000). Efficacy of long-term lamivudine monotherapy in patients with hepatitis B e antigen-negative chronic hepatitis B.. Hepatology..

[7] Tassopoulos NC, Volpes R, Pastore G, Heathcote J, Buti M, Goldin RD (1999). Efficacy of lamivudine in patients with hepatitis B e antigen-negative/hepatitis B virus DNA-positive (precore mutant) chronic hepatitis B. Lamivudine Precore Mutant Study Group.. Hepatology..

[8] Villeneuve JP, Condreay LD, Willems B, Pomier-Layrargues G, Fenyves D, Bilodeau M (2000). Lamivudine treatment for decompensated cirrhosis resulting from chronic hepatitis B.. Hepatology..

[9] Rosini N, Mousse D, Spada C, Treitinger A (2003). Seroprevalence of HbsAg, Anti-HBc and anti-HCV in Southern Brazil, 1999-2001.. Braz J Infect Dis..

[10] Naing L, Winn T, Rusli B (2006). Practical issues in calculating the sample size for prevalence studies.. Arch Orofacial Sci..

[11] Mayfield D, McLeod G, Hall P (1974). The CAGE questionnaire: validation of a new alcoholism screening instrument.. Am J Psychiatry..

[12] (2011). [Population-Based Study of Prevalence of infections by Hepatitis A, B and C viruses in the Capital of Brazil]..

[13] [Estudo de Prevalência de Base Populacional das Infecções pelos vírus das Hepatites A, B e C nas Capitais do Brasil]. 2010; Available from: http://www.aids.gov.br/sites/default/files/anexos/publicacao/2010/50071/estudo_prevalencia_hepatites_pdf_26830.pdf

[14] Manno M, Camma C, Schepis F, Bassi F, Gelmini R, Giannini F (2004). Natural history of chronic HBV carriers in northern Italy: morbidity and mortality after 30 years.. Gastroenterology..

[15] Zacharakis GH, Koskinas J, Kotsiou S, Papoutselis M, Tzara F, Vafeiadis N (2005). Natural history of chronic HBV infection: a cohort study with up to 12 years follow-up in North Greece (part of the Interreg I-II/EC-project).. J Med Virol..

[16] Ozer A, Yakupogullari Y, Beytur A, Beytur L, Koroglu M, Salman F (2011). Risk factors of hepatitis B virus infection in Turkey: A population-based, case-control study: Risk Factors for HBV Infection.. Hepat Mon..

[17] Wang YB, Chen EQ, Cui YL, Zeng L, Wang YJ, Tang H (2011). Seroprevalence of hepatitis B virus markers in individuals for physical examination in West China Hospital, China.. Eur Rev Med Pharmacol Sci..

[18] Sheikh NS, Sheikh AS, Sheikh AA, Yahya S, Lateef M (2011). Sero-prevalence of hepatitis B virus infection in Balochistan Province of Pakistan.. Saudi J Gastroenterol..

[19] Zhang H, Li Q, Sun J, Wang C, Gu Q, Feng X (2011). Seroprevalence and risk factors for hepatitis B infection in an adult population in Northeast China.. Int J Med Sci..

[20] Meffre C, Le Strat Y, Delarocque-Astagneau E, Dubois F, Antona D, Lemasson JM (2010). Prevalence of hepatitis B and hepatitis C virus infections in France in 2004: social factors are important predictors after adjusting for known risk factors.. J Med Virol..

[21] Lewis-Ximenez LL, do OK, Ginuino CF, Silva JC, Schatzmayr HG, Stuver S (2002). Risk factors for hepatitis B virus infection in Rio de Janeiro, Brazil.. BMC Public Health..

[22] Da Ros CT, Schmitt Cda S (2008). Global epidemiology of sexually transmitted diseases.. Asian J Androl..

[23] Torres JR (1996). Hepatitis B and hepatitis delta virus infection in South America.. Gut..

[24] McQuillan GM, Coleman PJ, Kruszon-Moran D, Moyer LA, Lambert SB, Margolis HS (1999). Prevalence of hepatitis B virus infection in the United States: the National Health and Nutrition Examination Surveys, 1976 through 1994.. Am J Public Health..

[25] Nuchprayoon T, Chumnijarakij T (1992). Risk factors for hepatitis B carrier status among blood donors of the National Blood Center, Thai Red Cross Society.. Southeast Asian J Trop Med Public Health..

[26] Nascimento MC, Mayaud P, Sabino EC, Torres KL, Franceschi S (2008). Prevalence of hepatitis B and C serological markers among first-time blood donors in Brazil: a multi-center serosurvey.. J Med Virol..

[27] Reynolds L, McKee M (2008). Possible risks of transmission of bloodborne infection via acupuncture needles in Guizhou province, southwest China.. J Altern Complement Med..

[28] Nguyen VT, McLaws ML, Dore GJ (2007). Highly endemic hepatitis B infection in rural Vietnam.. J Gastroenterol Hepatol..

[29] Yamashita H, Tsukayama H, White AR, Tanno Y, Sugishita C, Ernst E (2001). Systematic review of adverse events following acupuncture: the Japanese literature.. Complement Ther Med..

[30] Chen CJ, Wang LY, Yu MW (2000). Epidemiology of hepatitis B virus infection in the Asia-Pacific region.. J Gastroenterol Hepatol..

[31] Walsh B, Maguire H, Carrington D (1999). Outbreak of hepatitis B in an acupuncture clinic.. Commun Dis Public Health..

[32] Beasley RP, Hwang LY, Lin CC, Ko YC, Twu SJ (1983). Incidence of hepatitis among students at a university in Taiwan.. Am J Epidemiol..

[33] Phoon WO, Fong NP, Lee J (1988). History of blood transfusion, tattooing, acupuncture and risk of hepatitis B surface antigenaemia among Chinese men in Singapore.. Am J Public Health..

[34] Levin KA (2006). Study design IV. Cohort studies.. Evid Based Dent..

[35] Levin KA (2006). Study design III: Cross-sectional studies.. Evid Based Dent..

